# An Inguinal Hernia Radically Cured by Scarifying the Neck of the Sack

**Published:** 1847-06

**Authors:** G. N. Fitch

**Affiliations:** Professor of Institutes and Practice in Rush Medical College, Chicago, Illinois


ARTICLE II.

*An Inguinal Hernia radically cured by scarifying the Neck of the Sack.* By G. N. Fitch, M. D., Professor of Institutes and Practice in Rush Medical College, Chicago, Illinois.

James W., a farmer, aged 35 years, had an ancient scrotal hernia, of large size. On the 20th December, 1845, it became strangulated. My friend, Dr. A. B. Buchanan, of this place, (Logansport, Ind.,) visited him and left no exertion untried, for the space of ten hours, to remedy it, but in vain. The doctor then requested me to operate. No difficulty was experienced in the operation other than what arose after the division of the stricture from the reduction of a vast mass of intestine. Knowing the tumor would doubtless return after his recovery from the operation, and seriously interfere, as it had heretofore done, with his labor, his only means of subsisting himself and family, I determined to adopt Richter's advice (*Traite des Hernies*, p. 191), and scarify the neck of the sack to procure the adhesion of its sides. Accordingly a probe pointed bistoury was carried through it, by which it was freely scarified in every direction. Considerable peritoneal inflammation followed, but little, if any more, however, than would have followed the operation without the scarification. It yielded readily to appropriate treatment. Complete obliteration of the neck of the sack was obtained; as up to this time (March, 1847), there has been no appearance of the hernia, although the patient has used the utmost physical exertions incident to his laborious occupation in a new and heavily timbered country.

This is the first case of the kind in which I have followed

Richter's advice; but if cases present themselves requiring operation for strangulated hernia, it most assuredly will not be the last. Indeed I know of no good reason why so mild a means of radically curing this, at all times, troublesome and occasionally dangerous accident, is not generally resorted to. The most that can be urged against it is its occasional failure—but what surgical operation is not liable to the same objection?

